# Differing response properties of cervical and ocular vestibular evoked myogenic potentials evoked by air-conducted stimulation

**DOI:** 10.1016/j.clinph.2013.11.001

**Published:** 2014-06

**Authors:** Danielle L. Dennis, Sendhil Govender, Peggy Chen, Neil P. McAngus Todd, James G. Colebatch

**Affiliations:** a Prince of Wales Clinical School and Neuroscience Research Australia, University of New South Wales, Randwick, Sydney, NSW 2031, Australia; b Faculty of Life Science, University of Manchester, Manchester M13 9PL, UK

**Keywords:** oVEMP, cVEMP, Myogenic potentials, Superior canal dehiscence, Vestibular reflexes

## Abstract

**Objective:**

To determine the amplitude changes of vestibular evoked myogenic potentials (VEMPs) recorded simultaneously from the neck (cVEMPs) and eyes (oVEMPs) in response to 500 Hz, 2 ms air-conducted sound pips over a 30 dB range.

**Methods:**

Fifteen healthy volunteers (mean age 29, range 18–57 years old) and one patient with unilateral superior canal dehiscence (SCD) were studied. The stimulus was reduced in increments to 105 dB pSPL for the normals (81 dB pSPL for the SCD patient). A statistical criterion was used to detect responses.

**Results:**

Ipsilateral (i-p13/n23) and contralateral (c-n12/p24/n30) peaks for the cVEMP montage and contralateral (c-n10/p16/n21) and ipsilateral (i-n13) peaks for the oVEMP montage were present for the baseline intensity. For the lowest intensity, 6/15 subjects had responses for the i-p13 cVEMP potential and 4/15 had c-n10 oVEMP responses. The SCD patient showed larger responses for nearly all intensities. The cVEMP potentials were generally well fitted by a power law relationship, but the oVEMP c-n10, p16 and n21 potentials showed a significant increase in gradient for the higher intensities.

**Conclusion:**

Most oVEMP and cVEMP responses follow a power law relationship but crossed oVEMP responses showed a change in gradient above a threshold.

**Significance:**

The pattern of response to AC stimulation may be a property of the pathways underlying the potentials.

## Highlights

• cVEMPs and oVEMPS were recorded simultaneously from 15 healthy volunteers and 1 patient with superior canal dehiscence (SCD) using air conducted (AC) sound over a 30 dB range.
• The SCD patient had larger amplitude responses at all intensities except for the cVEMP at the loudest intensity.
• Whilst the cVEMP p13/n23 response was well fitted by a power law relationship the oVEMP n10/p16 response showed a change in gradient for the louder intensities and this may relate to differences in the pathways responsible.

## 1 Introduction

The vestibular apparatus has strong connectivity to both the eyes and neck mediating the vestibulo–ocular and vestibulocollic reflexes. Developments in vestibular research have given rise to non-invasive methods for assessment of these pathways by means of short latency evoked responses in the target muscles. Originally recorded over the sternocleidomastoid (SCM) muscles, the earliest response was termed a vestibular evoked myogenic potential (or VEMP) (Colebatch et al., 1994). These potentials are now commonly referred to as a cervical VEMP (CVEMP or cVEMP). A subsequently discovered myogenic response in peri-ocular locations was termed by analogy ocular VEMPs (OVEMPs or oVEMPs: Rosengren et al., 2005, Todd et al., 2007). Both recording sites are characterised by a series of short latency positive and negative waves which occur both ipsilaterally and contralaterally to a monaural air-conducted (AC) stimulus. For the cVEMP montage these include the ipsilateral p13, n23 (or i-p13/n23) and contralateral n12, p24 and n30 peaks (or c-n12/p24/n30), and for the oVEMP montage these are the contralateral n10, p16, n21 (or c-n10/p16/n21) and ipsilateral n13 peaks (or i-n13). Only the earlier potentials have been firmly established as being vestibular-dependent and, for the cVEMP montage in particular, the later peaks are unlikely to be of vestibular origin (Colebatch et al., 1994). VEMPs have proven to have useful diagnostic applications as well as providing a tool to investigate the properties of the human vestibular system (see Rosengren et al., 2010 for review).

It is generally agreed that VEMPs when activated by acoustic stimulation are a manifestation of the otolith-ocular or otolith-collic pathways but different modes of acoustic stimulation may produce different patterns of end-organ activation (Todd et al., 2007). There is evidence that mid-frequency AC sound stimulation (best frequency 500–1000 Hz) may be selective for the saccule, whilst low-frequency vibration of the head (best frequency 80–100 Hz) appears to be more selective for the utricle, especially if the direction of vibration is aligned within the plane of morphological polarisation of utricular hair-cells (Todd et al., 2008a, Todd et al., 2008b, Todd et al., 2009). Recent work by Zhang et al., 2011, Zhang et al., 2012 has provided evidence that both sound and vibration may produce distinct resonances at about 100 and 500 Hz, suggestive that the two resonance peaks are not specific to the two modes of stimulation, but to the different dynamic responses of the vestibular end organs. The matter remains controversial, however. Whilst the saccule has been shown to be responsive to acoustic stimuli (McCue and Guinan, 1997, Murofushi and Curthoys, 1997), the projections to the eyes have been reported to be weak (Isu et al., 2000) and the utricle has been proposed as an alternative source of the AC oVEMP (Curthoys, 2010). At this stage there is consensus that the responsible fibers are likely to arise from the otolith organs and travel via the superior vestibular nerve (e.g., Govender et al., 2011).

A fundamental property of any reflex is the input–output relationship – how the reflex response varies as a consequence of changes in the afferent input. An early study of the cVEMP identified the adequate air-conducted stimulus as being of high intensity, with larger responses occurring with higher stimulus intensities (Colebatch et al., 1994). Lim et al. (1995) reported a linear relationship between click intensity, measured in decibels (dB), and reflex cVEMP amplitude. No similar study has been performed for the oVEMP using AC stimuli, although Todd et al. (2008b) showed that low-frequency vibration-evoked oVEMPs followed a power-law relationship. The present study was designed to explore systematically the behaviour of the cVEMP and oVEMP reflexes and associated potentials to changes in stimulus amplitude whilst controlling for the effects of background activation. Our objective was to determine whether the relationship between intensity and reflex amplitude was the same for the different peaks recorded using the cVEMP and oVEMP montages and, more specifically, between the early cVEMP and oVEMP potentials, a finding that might be expected if both arose from the same receptor. A possible complicating factor is saturation of the cVEMP, which is known to be an inhibitory reflex (Colebatch and Rothwell, 2004). In addition, we wished to compare the thresholds for the responses as this might also indicate whether the same end organ was likely to generate both. One patient with superior canal dehiscence (SCD: Minor et al., 1998) was studied to compare with our findings in healthy subjects.

## 2 Methods

### 2.1 Subjects

Fifteen healthy adults aged 18–57 with no history of vestibular dysfunction participated in this study. Eleven subjects were tested at Prince of Wales Hospital, Sydney (7 men, 4 women; mean age 29 ± 14 years) and 4 subjects at University of Manchester (1 man, 3 women; mean age 31 ± 15 years). One patient with unilateral superior canal dehiscence (SCD) also participated (female; age 50 years) and was tested in Sydney. Dehiscence of the left superior canal had been previously confirmed in this patient using high-resolution CT imaging of the temporal bone and VEMP testing. Subjects gave written consent according to the Declaration of Helsinki before the experiment and the study was approved by the local ethics committees in Sydney and Manchester.

### 2.2 Stimuli

Stimuli were generated using custom software and a CED laboratory interface (1401plus, Cambridge Electronic Design, Cambridge, UK), and signal amplification was achieved using a custom amplifier. Subjects were presented with sinusoidal 500 Hz, 2 ms tone bursts (0 ms rise and fall) at a rate of ∼5 Hz. Stimuli were delivered using audiometric headphones (TDH 49, Telephonics Corp., Farmingdale, USA). The output was calibrated using a type 4192 pressure field microphone with a 4153 artificial ear and a 2260 sound level meter (Brűel & Kjær, Naerum, Denmark). The stimulus polarity was alternated to reduce stimulus artefact.

### 2.3 cVEMP and oVEMP montages

**Fig. 1:**
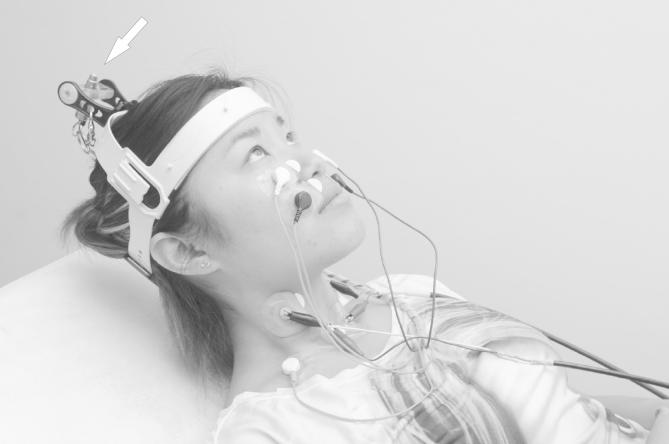
The experimental setup used to record cVEMPs and oVEMPs simultaneously in healthy subjects. As shown, the subjects lifted their heads to activate the neck muscles and a laser light (white arrow) was used to direct the subjects gaze to a projected red spot to keep gaze elevation constant despite changes in head position during head lifting. The level of tonic neck muscle activity was used to set the level of head elevation.

Electromyographic (EMG) activity was recorded simultaneously from the SCMs and below the eyes using self-adhesive Ag/AgCl electrodes (Cleartrace 1700-030, Conmed Corp., NY, USA). AC cVEMPs and oVEMPs obtained concurrently or separately yield the same results (Chou et al., 2009), and we have employed the simultaneous recording technique to shorten the procedure and to ensure the same conditions were applied to both reflexes. For the cVEMP montage, the active recording electrodes were placed on the upper third of the muscle belly and the reference electrodes on the sternal end of the clavicles. An earth electrode was placed above the lateral third of the clavicle. Subjects reclined to ∼30 degrees above horizontal and were required to lift their heads to activate the SCM muscles for the duration of the recording. For the oVEMP montage, electrodes were placed on the orbital margin inferior to both eyes and reference electrodes were positioned approximately 3 cm below them. A custom-made headband was used to secure a small laser pointer that projected a red spot onto the ceiling (Fig. 1). The pointer was positioned to produce an elevated gaze of ∼30 degrees for each subject and this was used as a constant point of reference for eye elevation regardless of slight changes in head position. Amplitudes were measured from the extraocular muscles both contralateral and ipsilateral to the stimulated ear. EMG was recorded for both the cVEMP and oVEMP montages from 20 ms before to 100 ms after stimulus onset and averaged over 200–250 individual trials using SIGNAL software (version 3, Cambridge Electronic Design, Cambridge, UK). Peaks were named using polarity and mean latency. For clarity, as we have analysed a number of peaks for both recording sites, we have used the prefix i- or c- when referring to a peak ipsilateral or contralateral to the stimulus. For the SCD patient, fewer individual trials were conducted at the high intensities (30–100) due to the response being easily detected and also to minimise patient discomfort.

### 2.4 Stimulus protocol

Each subject was stimulated in one ear which was chosen using a pseudo-randomised approach (11 on the left and 4 on the right). The SCD patient was stimulated using her left (affected) ear. Electrode impedance was maintained below 10 kΩ before recordings commenced. Intensity values are expressed as dB peak sound pressure level (pSPL) and the intensity used initially was 135 dB. Stimulus intensity was then decreased to 129 dB, and successively reduced in 3 dB steps thereafter, with 105 dB being the lowest intensity recorded for all subjects. The order was reversed for one subject (starting at 105 dB and then increasing intensities) and for another subject testing began at 114 dB, increased in 3 dB increments to 129 dB, and then finished with 111, 108, and 105 dB. Some subjects were not tested at 126 and 120 dB (*n *= 2) and 135 dB (*n *= 1). Recordings were repeated for all intensities for one subject, from 123 dB and below for seven subjects, from 120 dB and below for two subjects, and from 117 dB onwards for four subjects. One subject had repeat recordings for intensities 123, 120 and 117 dB only. A grand average record was made for each intensity but measurements of all individual recordings were also made (see below).

The recording protocol for the SCD patient was the same as for the healthy subjects but recordings at lower intensities were added as responses were still clearly present (102, 99, 93, 87 and 81 dB). Repeat recordings for the SCD patient were made from 93 dB and below. For all subjects the initial and the repeat recording of intensities were checked offline for reproducibility of peaks within the subjects and averaged to produce a single file for that intensity.

### 2.5 Data analysis

A set criterion of 2.5 standard deviations (s.d.) above or below the mean prestimulus activity was used to determine objectively whether a response was present for any given stimulus level. The latencies of any significant peak also had to be appropriate. Amplitudes were measured where the peaks were above the significance criterion. When they were not, to avoid bias, the amplitude value at the average latency for the peak was used. Correlations between peak amplitudes were performed for group-averaged amplitude values. Latencies were only measured for significant peaks. Signal-to-noise ratio (SNR) was defined as the ratio of the peak amplitude in question to the prestimulus standard deviation. The prestimulus standard deviation was measured for the lowest three intensities (111, 108, and 105 dB) as a guide to the sensitivity of our method. A threshold for each peak was determined for all subjects. We allowed a peak to fail to reach significance in a single intensity trial if it returned for at least the next lowest intensity (either −6 or −3 dB). Subjects with no responses to the loudest and second-loudest stimuli were assumed to have a threshold of 141 dB for the missing peaks. The overall thresholds for each subject were determined based on the c-n10 for the oVEMP, and for the cVEMP based on the i-p13.

Linear regressions were then calculated using the reflex amplitude (in μV) against intensity (in dB) and then for log-transformed reflex amplitudes (using a 1 μV reference). The second approach was based upon the model of Todd et al. (2008b), namely:

$$V(s)={\mathrm{ks}}^{\beta }$$

where *V* is the response amplitude, *k* the scaling constant, *s* the intensity (Pa) and *β* is the power law parameter. When transformed to a log–log plot or dB vs dB plot in our case, *β* is given as the slope of the linear fit. We defined dB as 20 ∗ log_10_ (ratio to reference) for both sound intensity and reflex amplitude. Regression analysis was performed for raw and log transformed averaged amplitude values for all subjects’ i-p13, n23, c-n12, c-p24, and c-n30 peaks (cVEMP montage) and c-n10, c-p16, c-n21 and i-n13 peaks (oVEMP montage) from 105 to 135 dB. Departures from the linear fit were determined by performing a quadratic regression and testing the significance of the reduction in residual error due to the quadratic component (Snedecor and Cochran, 1989). Where the quadratic term was significant, separate regressions were performed for the lower and upper intensities. Regression gradients were compared using the method of Gardner and Altman (1989). The SCD patient’s data was regressed from 105 to 135 dB for the i-p13-n23 and c-n10-p16 peaks.

## 3 Results

Comparison of data between laboratories showed only one peak amplitude difference and two peak latency differences at baseline (oVEMP: c-p16, *P *= 0.039; oVEMP c-n10, *P *= 0.048; cVEMP c-p24, *P *= 0.011, respectively; not significant after Bonferroni correction) and all analyses were conducted on the combined data. An ANOVA showed a significant side to side difference for a single peak and stimulus condition only (c-p16 oVEMP, left larger) thus channels for the subjects stimulated on the right were exchanged so that a grand average could be made, with the side of stimulation effectively being on the left in all cases. There was no significant difference in background SCM EMG activity (114.2 ± 37.6 μV) across intensities (*F*_9,270_ = 0.2, *P *= 0.989). The prestimulus standard deviation was 3.0 μV for the grand averaged cVEMP and 0.1 μV for the grand averaged oVEMP. For the individual subjects these values were higher, being on average 6.4 μV for the cVEMP to 0.3 μV for the oVEMP. We tested the reliability of our significance criterion by measuring how many of the averaged trials showed a peak above our criteria during the prestimulus record. Of 290 averaged recordings for each modality, 4.8% of cVEMPs, and 18% of oVEMPS showed a peak exceeding the 2.5 times standard deviation criterion during the prestimulus interval.

### 3.1 Response amplitudes-grand average record

**Fig. 2:**
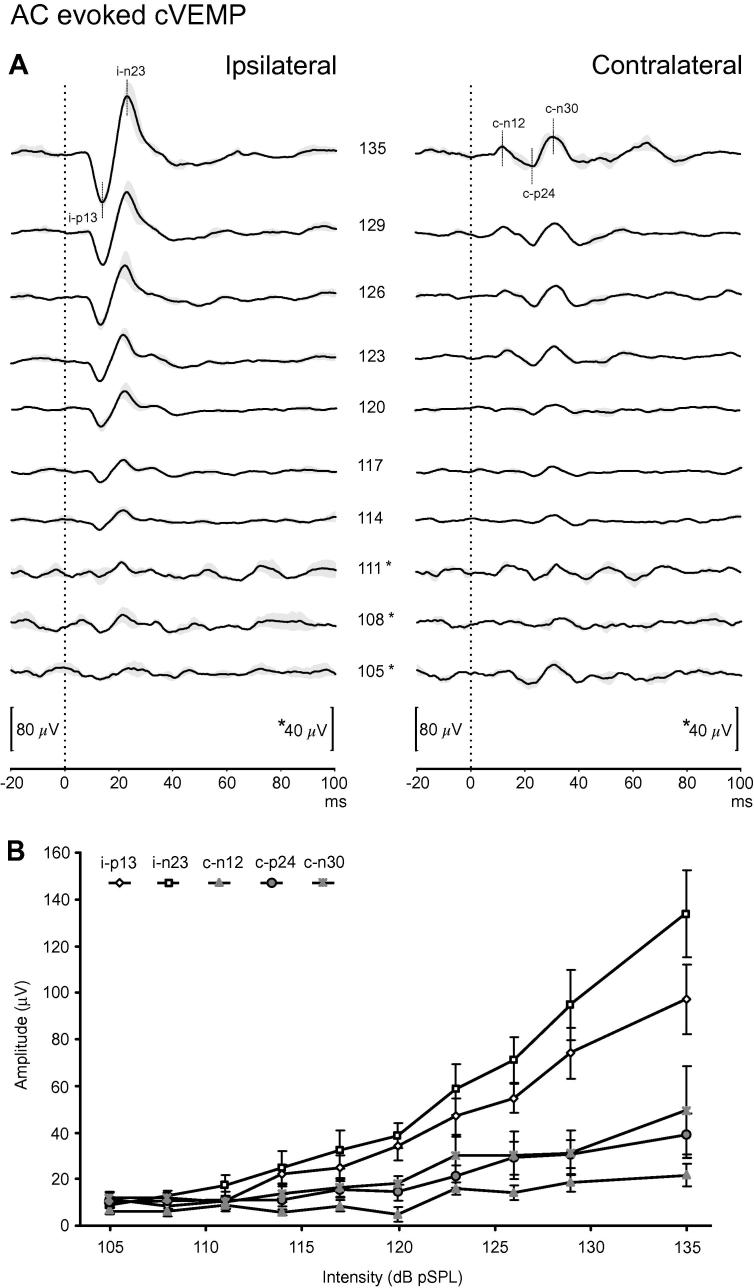
Upper (A): grand mean traces of AC evoked cVEMPs (*n* = 15) at each intensity level. Values represent intensity in dB pSPL. Grey shading represents ± 1 SEM. The rate of decrease in response amplitude with the lowest intensities was more marked for the initial contralateral peak than for p13 and n23 peaks from the ipsilateral SCM. Lower (B): Averaged amplitudes plus SEM from individual subjects showing that the ipsilateral (i-p13, i-n23) and contralateral (c-n12, c-p24, c-n30) responses to changes in stimulus intensity were not linear. Whilst data is plotted for increasing intensity, recordings were usually made with sequentially decreasing intensities.

**Fig. 3:**
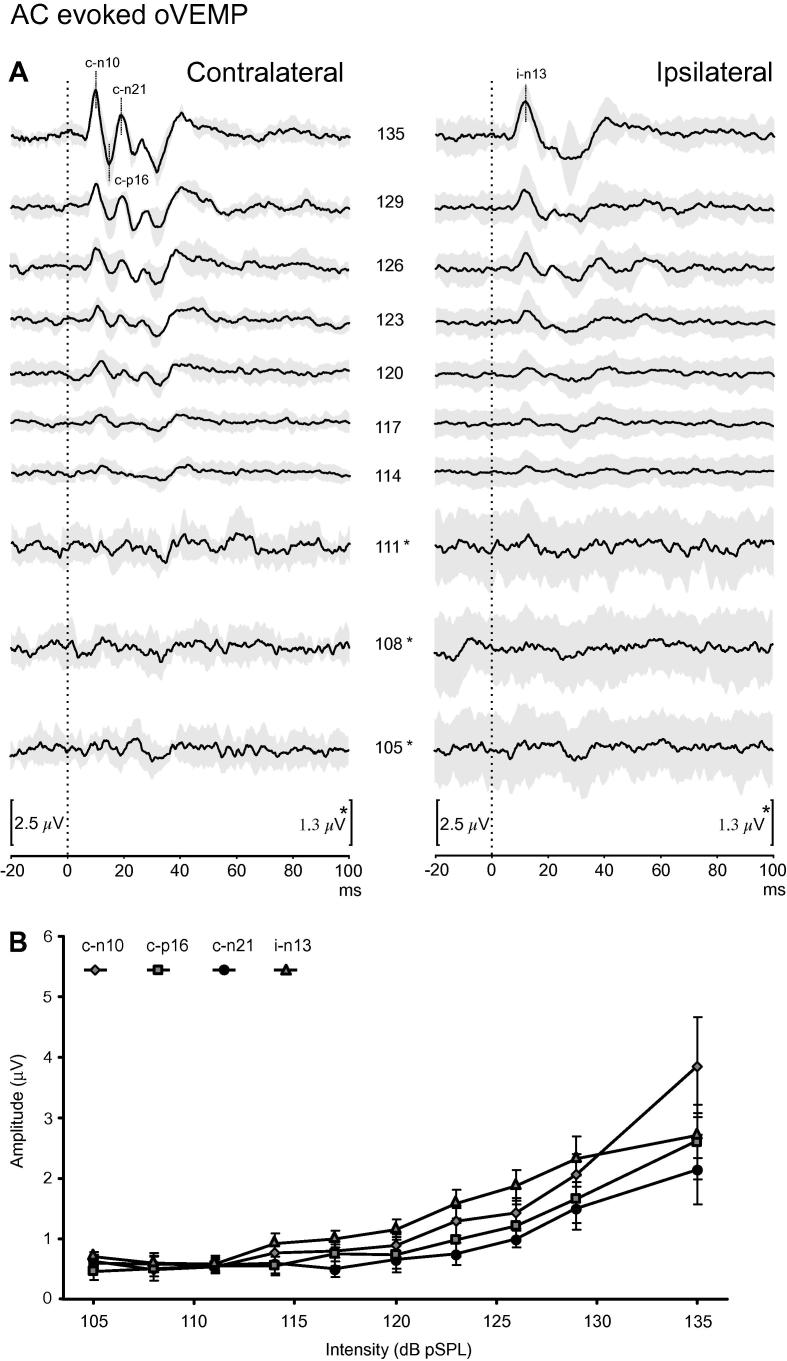
Upper (A): grand mean traces of AC evoked oVEMPs (*n* = 15) for each intensity level. Values represent intensity in dB pSPL. Grey shading represents ± 1 SEM. Peaks were recorded from beneath the contralateral (c-n10, c-p16, c-n21) and ipsilateral eyes (i-n13). Note increased gain for lowest stimulus intensities. Lower (B): plot of the raw amplitudes and SEM of the various peaks measured from individual subjects in response to decreasing stimulus intensity.

Grand average traces are shown in Fig. 2, Fig. 3 and represent the mean of approximately 3000 individual trials. The baseline recordings indicated that multiple peaks were significant by our criteria. For the cVEMP montage these were the i-p13, n23 and the c-n12, p24, n30 peaks. For the oVEMP montage, the c-n10, p16, n21 and i-n13 peaks were above our criterion. These peaks were therefore measured for this and the remaining intensities in all subjects. The SNR for the baseline peaks varied from 32 (i-n23 cVEMP) to 3.0 (c-n12 cVEMP; Table 1). Measured using our significance criterion for the grand averaged recording of the cVEMP, the i-n23 had the lowest threshold (108 dB) and the c-n12 had the highest (123 dB). Using the grand averaged oVEMP record, the i-n13 had the lowest threshold (111 dB) whilst the c-p16 and c-n21 (120 dB) had the highest (Table 1).

**Table 1:** Averaged peaks values for baseline intensity stimulation.

	cVEMP montage					oVEMP montage			
	i-p13	i-n23	c-n12	c-p24	c-n30	c-n10	c-p16	c-n21	i-n13
Amplitude (μV)	105	119	13.4	28.9	33.5	2.7	1.5	1.2	2.4
SD (μV)	3.8	3.8	4.4	4.4	4.4	0.1	0.1	0.1	0.1
SNR	28	32	3.0	6.5	7.6	21	11	9.1	17
Grand average threshold (dB pSPL)	114	108	123	117	111	114	120	120	111
Population average threshold (dB pSPL)	114.4 ± 5.2	113.2 ± 7.5	126.6 ± 5.1	120.2 ± 7.6	117.5 ± 8.3	121.5 ± 7.9	119.3 ± 6.9	120.8 ± 7.8	114.8 ± 5.7

Measurements made for the first 4 rows using grand averaged recordings.

SD, standard deviation of prestimulus recording (noise); SNR, signal to noise ratio.

Population average refers to the average of individual subject measurements.

### 3.2 Response amplitudes and thresholds-individual measurements

The mean baseline amplitudes obtained for the individual measurements of the cVEMP and related peaks were 96.9 μV (i-p13), 133.1 μV (i-n23), 21.9 μV (c-n12), 39.1 μV (c-p24) and 49.5 μV (c-n30: Supplementary Table S1). For the oVEMP the mean baseline amplitudes were 3.8 μV (c-n10), 2.6 μV (c-p16), 2.1 μV (c-n21) and 2.7 μV (i-n13: Supplementary Table S2). At the baseline intensity, the number of subjects (of the 14 tested) with responses meeting our criteria varied, for the cVEMP, the responders were: 14 (i-p13, i-n23), 6 (c-n12), 10 (c-p24) and 10 (c-n30) and for the oVEMP 13 (c-n10), 11 (c-p16), 10 (c-n21) and 13 (i-n13). The proportion of subjects showing responses fell with reducing intensity but even for the least intense stimulus, 6 of 15 subjects still showed a significant cVEMP i-p13 peak and 4 of 15 showed an oVEMP c-n10 peak (Supplementary Tables S1 and S2). The two subjects who were tested with different intensity order showed responses similar to those of the other subjects.

The mean thresholds measured from the individual data were mostly similar to those using the grand average (Table 1). For the cVEMP montage, the i-n23 had the lowest threshold overall (113.2 dB) and the c-n12 the highest (126.6 dB). One subject had responses at every intensity for the i-p13 peak whilst two other subjects had similar responses for the n23 peak. For the oVEMP montage, the c-n10, p16 and n21 all had mean thresholds around 120 dB. One subject had consistent responses (135–105 dB) for both the c-n10 and c-p16 peaks, whilst one subject had the same for the i-n13. ANOVA analysis of individual thresholds, with Bonferroni correction, showed that the cVEMP c-n12 had a significantly higher threshold than the i-p13 and i-n23 responses and for the oVEMP i-n13 response (*P *< 0.001 for all). The oVEMP c-n10 response had a significantly higher threshold than the i-n13 response (*P *= 0.0011). There was a trend for the cVEMP i-p13 response to have a lower threshold than the oVEMP c-n10 but this did not reach significance after correction (*P *= 0.003, uncorrected).

### 3.3 The relationship between amplitude and intensity

**Fig. 4:**
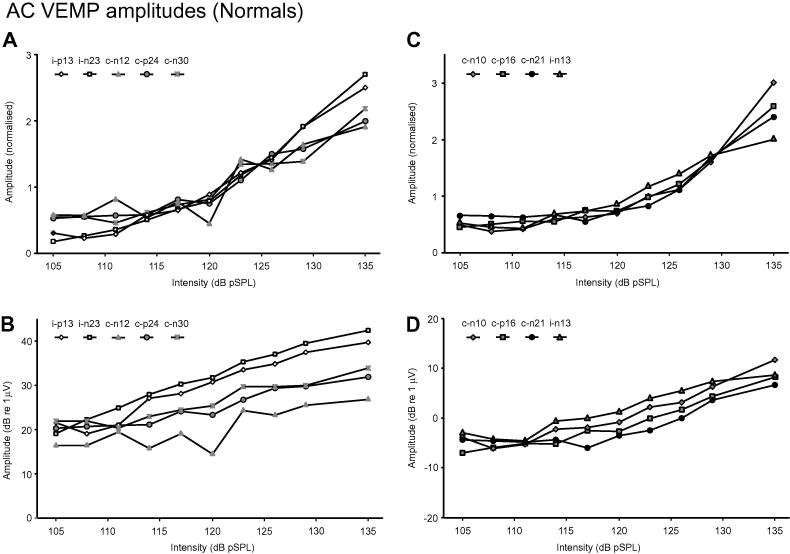
cVEMP (A and B) and oVEMP (C and D) amplitudes plotted against stimulus intensity. For the upper half of the figure, response amplitudes have been rescaled to a value of 1 for the average values obtained. Both the cVEMP (A) and oVEMP (C) responses show curvilinear responses to intensity. In the lower half, the data are plotted after the raw amplitudes were converted to dB (see text), resulting in a linear fit for most of the peaks studied.

The raw amplitudes versus sound intensity plots were curvilinear for all the potentials measured (Fig. 4A and C) and all showed highly significant quadratic components. The logarithmically-transformed amplitudes were more linear and were regressed against sound intensity (both measured in dB: Fig. 4B and D).

**Fig. 5:**
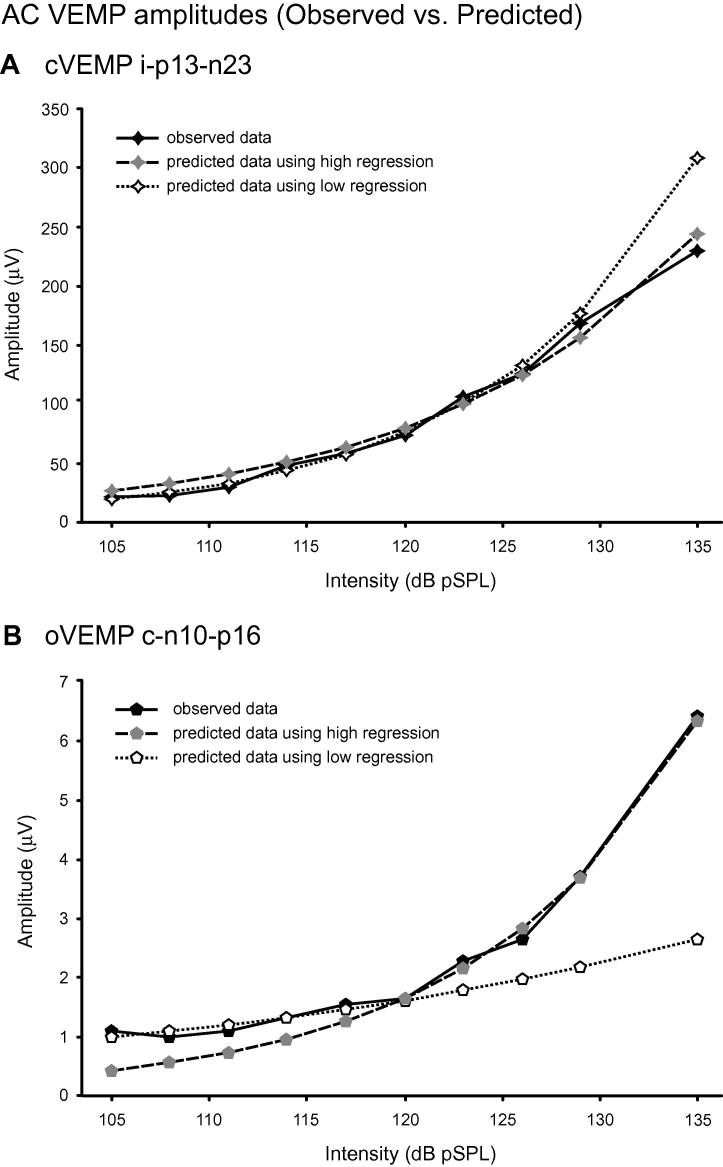
Part A shows the observed and predicted amplitudes of the group-averaged i-p13-n23 responses for the cVEMP against intensity, using regressions based upon the lower and higher intensities. Both regressions give predicted curves that fit the observed data well. Part B shows similar data using the oVEMP c-n10-p16 responses. The initial regression showed the presence of a significant quadratic component (i.e., non-linear curvature) and whilst the fits using the points below 120 dB and for 120 dB and above both match the observed data well for these intensities, they have significantly different gradients (using the log-transformed reflex amplitudes). Corresponding to this, the curvature increases for stimuli over 120 dB pSPL.

For the cVEMP peaks, the gradients of the regressions ranged from 0.379 to 0.787 and for the oVEMP peaks, from 0.374 to 0.553 (Table 2). Testing the transformed amplitudes using the quadratic fit showed different findings for the cVEMP and oVEMP peaks. For the cVEMP, only the n23 showed a significant improvement in fit with the addition of a (negative) quadratic term, whilst the other 4 peaks were fitted well using a linear relationship alone. In contrast, for the oVEMP, only the i-n13 was well fitted with the linear regression alone whilst the other peaks showed significant improvements in fit with a positive quadratic term, indicating a concave (increasing) gradient with increasing intensity. Comparing the regressions fitted using the lower and upper 5 intensities confirmed the findings with the quadratic regression (Table 3). The cVEMP i-n23 showed a significant reduction in gradient for the higher intensities. The oVEMP c-n10, c-p16 and c-n21 potentials all showed significant increases in gradients for the higher intensities. Fig. 5 shows the behaviour of the cVEMP i-p13-n23 potential compared to that of the oVEMP c-n10-p16 potentials for the lower and higher intensities. The oVEMP amplitudes for the lower intensities were larger than expected from the relationship shown for the higher intensity stimuli.

**Table 2:** cVEMP and oVEMP regressions for log transformed data.

Peak	cVEMP montage					oVEMP montage			
	i-p13	i-n23	c-n12	c-p24	i-n30	c-n10	c-p16	c-n21	i-n13
Gradient (SCD)	0.730 (0.192)	0.787 (−0.032^a^)	0.379	0.430	0.448	0.553 (0.588)	0.498 (0.534)	0.374	0.479
*r*^2^	0.950	0.990	0.658	0.931	0.909	0.906	0.946	0.746	0.933
*F*(1,7)⁎	0.225	20.7⁎⁎	1.19	1.97	1.88	20.5⁎⁎	28.3⁎⁎	33.7⁎⁎	0.45

Gradients for 20× log (raw amplitude) vs intensity.

Gradients for normal subjects and SCD patient calculated using 135–105 dB pSPL data.

Gradient values for SCD patient indicated in brackets.

a Not significantly different from 0.

⁎ *F* value for error reduction for quadratic regression (Snedecor and Cochran, 1989).

⁎⁎ *P* < 0.01.

**Table 3:** cVEMP and oVEMP regressions for log transformed data for low and high intensities.

Peak	cVEMP montage		oVEMP montage					
	i-n23		c-n10		c-p16		c-n21	
	Low	High	Low	High	Low	High	Low	High
Gradient	0.932	0.694	0.265	0.824	0.319	0.725	−0.095	0.730
*r*^2^	0.997	0.972	0.459	0.985	0.854	0.997	0.489	0.974
*P* (vs 0)	0.000⁎⁎	0.002⁎	0.209	0.001⁎	0.025⁎	0.000⁎⁎	0.196	0.002⁎
*P* (Low vs High)	0.025⁎		0.015⁎		0.002⁎		0.000⁎	

Gradients for peaks with a significant quadratic element (see text).

Gradients for normal subjects calculated using AC stimuli 105–117 dB (“Low”) and 120–135 dB (“High”).

⁎ Probabilities <0.05.

⁎⁎ Probabilities ⩽0.0001.

For individual subjects, the gradients for the transformed cVEMP i-p13-n23 response varied from 0.57 to 0.99 (*n* = 14, all significantly >0, *P *< 0.006) and for the oVEMP c-n10-p16 response from 0.09 to 1.07 (*n* = 6, 3 significantly >0, *P *< 0.042).

### 3.4 Response latencies

The mean latencies for the peaks recorded using the cVEMP montage showed no significant change with decreasing intensity (Supplementary Table S1: *F*_8–9,28–103_ = 2.2–0.2, *P *= 0.058–0.993). For the oVEMP, there was no significant change in response latency with decreasing intensity for either the c-p16 (Supplementary Table S2: *F*_9,46_ = 1.1, *P *= 0.404) or the c-n21 peaks (*F*_9,46_ = 1.9, *P *= 0.071). The c-n10 latency increased as the intensity decreased (*F*_9,62_ = 3.6, *P *= 0.001) with latencies at 126–108 dB being significantly longer than at 135 dB (*t*_14–20_ = 4.2–2.1, *P *= 0.001–0.046). Latencies for the i-n13 peak also increased as intensity decreased (*F*_9,85_ = 2.2, *P *= 0.034) with latencies at 105 dB being significantly longer than latencies at all intensities from 135 to 114 dB (*t*_4.3–18_ = 3.5–2.2, *P *= 0.006–0.047).

### 3.5 SCD subject

The SCD patient showed substantially larger responses than the normal subjects at all intensities for the oVEMP c-n10 and p16 peaks and for all but the highest intensities for the cVEMP i-p13 and n23 responses (Supplementary Tables S1 and S2), with saturation occurring for the patient for the cVEMP potential and possibly for the oVEMP at the most intense stimuli. For both the cVEMP i-p13 and oVEMP c-n10 the patient’s thresholds were 93 dB pSPL. The oVEMP c-n10/p16 responses were persistent from baseline down to 93 dB, as were responses for the cVEMP i-p13. The patient’s cVEMP i-p13-n23 gradient was significantly less than that of the normal subjects’ (0.095, *P* ≪ 0.001) whilst the oVEMP c-n10-p16 gradient lay within the normal range (0.557).

## 4 Discussion

We have used an objective measure of the presence or absence of a response for both the cVEMP and oVEMP montages and have examined their properties over a 30 dB range. Our findings complement the normative values reported by Rosengren et al. (2011) by defining the changes with intensity for cVEMPs and oVEMPs. Our baseline values are higher than those reported by Rosengren et al. (2011), probably due to the slightly more intense stimulus we used and the younger average age of our subjects. Lim et al. (1995) conclusion that raw cVEMP reflex amplitude was linearly related to stimulus intensity was based upon observations at only 3 intensities, a sample not adequate to detect the non-linearity of the relationship. McNerny and Burkard (2011) compared cVEMPs for AC and BC over a 30 dB range but reported the relationship was simply “monotonic” without further characterising it. Measuring responses with low intensity stimuli is difficult and requires consideration of signal to noise ratio (SNR). Todd et al. (2010) showed that the subjective detection of both cVEMPs and oVEMPs followed similar relations when plotted against SNR, with a steep increase in the proportion of true positives occurring with SNR of 2 and above. We tried to reduce the subjective element in determining the presence or absence of a response by making this determination using a statistical criterion. Our approach has demonstrated that some normal subjects can have (small) responses to relatively low intensity stimuli. In particular, some normal subjects can have responses to stimuli of 105 dB pSPL for both the i-p13 and n23 peaks of the cVEMP and the c-n10 peak of the oVEMP. A power law relationship implies no definite threshold but diminishingly small responses as the stimulus gets less intense. Any threshold determination therefore will be strongly affected by the number of trials averaged. Our estimates of threshold nevertheless were similar to previous reports for AC thresholds for cVEMPs and oVEMPs, with the cVEMP i-p13 threshold being on average 7.1 dB lower than that for the oVEMP c-n10 response using individual records. The cVEMP i-p13 and oVEMP c-n10 thresholds were closer however when measured using the grand average traces.

We have shown that the relationship for the potentials recorded with the cVEMP montage are well fitted using a logarithmic transformation of reflex amplitude and, in particular, the fits for the mean p13 and n23 potentials (in dB) had *r*^2^ values of over 0.95. The dB intensity measure for the stimulus is proportional to the energy in the waveform which Rosengren et al. (2009) have shown is an important determinant of p13-n23 cVEMP amplitude. The n23 response however did show evidence of significant (negative) curvature, probably due to saturation of the underlying inhibitory pathway (Colebatch and Rothwell, 2004). The average gradient for the two potentials, 0.76, implies that the cVEMP p13-n23 reflex amplitude increased by 2.4 times for a 10 dB increase in intensity over the range tested. The fit was not confined to peaks of proven vestibular origin as the i-n30 peak, which is likely to be of cochlear origin (Colebatch et al., 1994), was also well fitted (*r*^2^ = 0.91), albeit with a lower gradient.

Todd et al. (2008b) found that a power law relationship fitted the oVEMP responses evoked by head acceleration over a 50 dB range. They reported a gradient of 0.66, slightly higher than our findings for the oVEMP c-n10 and c-p16 peaks evoked by AC stimuli. In contrast to the peaks from the cVEMP montage, most of the oVEMP peaks showed a relationship which was not well fitted using a simple linear relationship even after logarithmic transformation. Nearly all showed a significant increase in the gradient of the relationship once the stimulus exceeded a certain level, the sole exception being the one ipsilateral (n13) response. It may be significant that it was the crossed pathways for the oVEMP which showed the apparent thresholds whereas the ipsilateral projections for both the oVEMP and cVEMP showed consistent behaviour throughout the range of stimuli presented. For the lower intensity levels, the average oVEMP peak amplitudes were all less than 1 μV ( = 0 dB). The amplitudes predicted from the relationship based upon more intense stimulation would have been very small and it is possible that, despite our bipolar recording montage, that other non-myogenic sources might be contributing to the contralateral potentials for the low stimulus intensities. For example, Todd et al. (2008c) reported that there were deep sources, possibly within the cerebellum, which were co-active with oVEMPs and auditory-evoked responses have been recorded from the cerebellum (Snider and Stowell, 1944). It may be that small responses recorded with the oVEMP montage do not originate solely from extraocular muscles, in contrast to what has been directly demonstrated for responses to intense stimuli (Weber et al., 2012).

When the oVEMP response was initially reported it was assumed that the AC-evoked response was likely to arise in the same way as the AC-evoked cVEMP and initially this appeared to be the case (Chihara et al., 2007). Todd et al. (2009) found similar tuning for AC-evoked cVEMPs and oVEMPs, with a broad peak between 400 and 800 Hz which they explained as likely to be a consequence of the resonance properties of the saccule. Conversely they found evidence of a lower resonant frequency, around 100 Hz, for what they took to be utricular responses, observations that they explained in terms of the structures of the two otoliths. Dissociations in the findings for AC-evoked cVEMPs and oVEMPs have been recognised to occur in vestibular neuritis, a condition with preferential involvement of the superior division of the vestibular nerve (Fetter and Dichgans, 1996). Typically the AC-evoked oVEMP is lost in this condition whereas the AC-evoked cVEMP is often less affected (Govender et al., 2011). The latter authors suggested that saccular fibres travelling in the superior division of the vestibular nerve might thus be responsible for evoking the oVEMP. Alternatively it has been proposed that the effects of AC stimuli are mediated by utricular fibres (Curthoys, 2010) because the saccular projection to extraocular muscles is weak when intracellular recordings have been made (Isu et al., 2000). A problem with accepting these intracellular findings as relevant to humans is that a crossed projection from the utricule to inferior oblique motoneurons, the proposed basis of the c-n10 potential, has not been demonstrable using these techniques in cats (Uchino et al., 1996). One explanation of our findings would be that the apparent oVEMP behaviour is indicative of recruitment of utricular afferents causing increase in the gradient of the responses. The behaviour of the cVEMP c-n12 response, which is consistent with a crossed utricular effect (Kushiro et al., 1999, Welgampola and Colebatch, 2001), might be expected to be a guide in this regard, but this peak had the lowest SNR of the group and cannot be relied upon too heavily. One reason to be cautious in attributing the threshold and change in gradient to recruitment of utricular afferents is the high gradients shown using the more intense stimuli for the oVEMPs recorded contralaterally. This implies involvement of afferents with a high affinity for the stimulus. The gradients are even higher than those for the p13 and n23 peaks of the cVEMP, peaks which may be taken to be indicative of the pattern of recruitment of saccular afferents. Alternatively, the change in gradient may simply be a property of the crossed pathway that mediates the responses. One way to resolve this issue may be to investigate the pattern of response to a stimulus which is more specific for utricular fibres, to see whether the gradient change is still present. Our findings about the differing properties of the cVEMP i-p13/n23 response and the oVEMP c-n10/p16 response might also be relevant to their differing responses to disease.

A power-law relationship clearly cannot continue as intensity increases. All reflexes, inhibitory or excitatory, will eventually saturate. It is likely that the underlying relationship is closer to sigmoidal and that our observations represent the behaviour of several of the reflexes before there is an inflection in the curve. In some patients this saturation was evident and in our series the cVEMP i-n23 potential gradient fell with increasing intensity. In SCD a greater proportion of the sound energy is diverted to the vestibular apparatus thus causing much more effective stimulation than in healthy subjects (Rosowski et al., 2004), including afferents arising from the superior canal (Aw et al., 2006, Rosengren et al., 2008). This condition illustrates the reflex changes occurring with relatively more intense stimuli. For the SCD patient, the thresholds for both reflexes were the same and lower than for all our normal subjects. For the cVEMP, where amplitude differences between SCD patients and healthy subjects are known to be less reliable using conventional intensities (Rosengren et al., 2008), our patient confirms that the greatest separation from normal values occurs using less intense stimuli (Brantberg and Verrecchia, 2012). In contrast, the separation for the oVEMP was large for our patient for nearly all intensities, including the loudest (Rosengren et al., 2008, Welgampola et al., 2008). More observations will be required using patients with SCD to determine the optimum level of stimulation for separation from normal responses.

## Appendix A Supplementary data

Supplementary Tables This document file contains Supplementary Tables S1 and S2.
