# Supplementary material for: Differing response properties of cervical and ocular vestibular evoked myogenic potentials evoked by air-conducted stimulation
Source: Clin Neurophysiol. 2014 Jun;125(6):1238–47. doi: 10.1016/j.clinph.2013.11.001 (PMC4032573; doi:10.1016/j.clinph.2013.11.001)
Supplement: Supplementary Tables — This document file contains Supplementary Tables S1 and S2. [file mmc1.doc]

**Supplementary Table S1.** cVEMP montage amplitude, latency and proportion of significant responses.

|  | | |  | *Intensity (dB p SPL)* | | | | | |
| --- | --- | --- | --- | --- | --- | --- | --- | --- | --- |
|  |  |  |  | *135* | *129* | *123* | *117* | *111* | *105* |
|  |  |  |  |  |  |  |  |  |  |
| *cVEMP peaks* | Amplitude  (µV) | i-p13 |  | 96.9 ± 55.6  *(125.3)* | 74.0 ± 41.7  *(98.6)* | 47.0 ± 30.6  *(115.2)* | 25.3 ± 19.7  *(103.7)* | 11.2 ± 14.3  *(95.4)* | 11.9 ± 11.6  *(98.1)* |
|  |  | i-n23 |  | 133.1 ± 69.9  *(48.8)* | 94.3 ± 58.1  *(112.8)* | 58.5 ± 41.7  *(122.4)* | 32.8 ± 31.6  *(114.6)* | 17.7 ± 17.6  *(95.2)* | 9.1 ± 12.7  *(91.5)* |
|  |  | c-n12 |  | 21.9 ± 18.2 | 18.9 ± 14.6 | 16.3 ± 10.1 | 9.0 ± 9.0 | 9.4 ± 10.9 | 6.6 ± 5.2 |
|  |  | c-p24 |  | 39.1 ± 35.3 | 30.9 ± 23.2 | 21.7 ± 18.7 | 16.0 ± 13.6 | 11.3 ± 13.7 | 10.5 ± 8.6 |
|  |  | c-n30 |  | 49.5 ± 70.4 | 31.5 ± 36.7 | 30.4 ± 31.0 | 16.7 ± 15.6 | 10.4 ± 10.0 | 12.5 ± 7.9 |
|  |  |  |  |  |  |  |  |  |  |
|  | Latency  (ms) | i-p13 |  | 13.7 ± 1.1 | 13.4 ± 1.0 | 13.4 ± 1.9 | 13.4 ± 2.4 | 13.6 ± 1.9 | 14.8 ± 2.9 |
|  |  | i-n23 |  | 22.1 ± 1.4 | 22.0 ± 1.7 | 21.9 ± 2.2 | 22.0 ± 2.6 | 22.2 ± 3.5 | 23.1 ± 2.8 |
|  |  | c-n12 |  | 13.5 ± 1.6 | 12.8 ± 2.2 | 13.9 ± 1.5 | 14.3 ± 1.3 | 15.3 ± 2.0 | 11.9 ± 1.3 |
|  |  | c-p24 |  | 22.2 ± 2.9 | 23.7 ± 0.8 | 24.0 ± 1.2 | 23.5 ± 2.3 | 24.5 ± 1.6 | 21.1 ± 2.6 |
|  |  | c-n30 |  | 30.9 ± 2.8 | 31.9 ± 2.4 | 31.6 ± 2.1 | 31.2 ± 1.7 | 32.1 ± 2.2 | 30.2 ± 1.4 |
|  |  |  |  |  |  |  |  |  |  |
|  | Proportion  of SR (%) | i-p13 |  | 14/14 (100) | 15/15 (100) | 13/15 (87) | 12/15 (80) | 4/15 (27) | 6/15 (40) |
|  |  | i-n23 |  | 14/14 (100) | 11/15 (73) | 15/15 (100) | 14/15 (93) | 10/15 (67) | 4/15 (27) |
|  |  | c-n12 |  | 6/14 (43) | 8/15 (53) | 5/15 (33) | 3/15 (20) | 3/15 (20) | 2/15 (13) |
|  |  | c-p24 |  | 10/14 (71) | 11/15 (73) | 8/15 (53) | 8/15 (53) | 6/15 (40) | 3/15 (20) |
|  |  | c-n30 |  | 10/14 (71) | 10/15 (67) | 10/15 (67) | 6/15 (40) | 3/15 (20) | 6/15 (40) |
|  |  |  |  |  |  |  |  |  |  |

Population averaged amplitudes and latencies (± s.d.) for cVEMP montage peaks under study. The lowermost rows show the proportion of subjects with significant responses (SR) at each stimulus intensity for the peaks contralateral (c) and ipsilateral (i) to the stimulus. Italicized values in backets indicate SCD amplitudes. Note: not all intensities studied are listed.

**Supplementray Table S2.** oVEMP montage amplitude, latency and proportion of significant responses.

|  | | |  | *Intensity (dB p SPL)* | | | | | |
| --- | --- | --- | --- | --- | --- | --- | --- | --- | --- |
|  |  |  |  | *135* | *129* | *123* | *117* | *111* | *105* |
|  |  |  |  |  |  |  |  |  |  |
| *oVEMP peaks* | Amplitude  (µV) | c-n10 |  | 3.8 ± 3.1  *(26.8)* | 2.1 ± 1.4  *(22.9)* | 1.3 ± 0.9  *(18.7)* | 0.8 ± 0.4  *(17.7)* | 0.5 ± 0.4  *(17.5)* | 0.6 ± 0.4  *(15.7)* |
|  |  | c-p16 |  | 2.6 ± 2.3  *(25.4)* | 1.7 ± 1.5  *(24.1)* | 1.0 ± 1.1  *(21.0)* | 0.8 ± 0.8  *(20.0)* | 0.6 ± 0.5  *(22.5)* | 0.5 ± 0.5  *(16.3)* |
|  |  | c-n21 |  | 2.1 ± 2.2 | 1.5 ± 1.4 | 0.8 ± 0.7 | 0.5 ± 0.5 | 0.6 ± 0.4 | 0.6 ± 0.4 |
|  |  | i-n13 |  | 2.7 ± 1.4 | 2.3 ± 1.5 | 1.6 ± 0.9 | 1.0 ± 0.5 | 0.6 ± 0.5 | 0.7 ± 0.3 |
|  |  |  |  |  |  |  |  |  |  |
|  | Latency  (ms) | c-n10 |  | 9.7 ± 1.3 | 10.2 ± 1.4 | 11.7 ± 1.4 | 12.4 ± 1.6 | 11.8 ± 2.3 | 11.8 ± 2.3 |
|  |  | c-p16 |  | 14.6 ± 1.4 | 16.0 ± 3.0 | 16.1 ± 2.6 | 16.3 ± 1.1 | 16.5 ± 1.3 | 18.6 ± 0.0 |
|  |  | c-n21 |  | 19.4 ± 1.3 | 19.6 ± 1.0 | 20.6 ± 2.7 | 20.5 ± 1.2 | 21.7 ± 3.8 | 22.6 ± 2.7 |
|  |  | i-n13 |  | 11.8 ±1.2 | 12.7 ± 3.3 | 13.5 ± 2.9 | 13.6 ± 2.9 | 12.6 ± 0.6 | 18.3 ± 4.1 |
|  |  |  |  |  |  |  |  |  |  |
|  | Proportion  of SR (%) | c-n10 |  | 13/14 (93) | 11/15 (73) | 10/15 (67) | 6/15 (40) | 3/15 (20) | 4/15 (27) |
|  |  | c-p16 |  | 11/14 (79) | 9/15 (60) | 7/15 (47) | 5/15 (33) | 4/15 (27) | 1/15 (7) |
|  |  | c-n21 |  | 10/14 (71) | 9/15 (60) | 7/15 (47) | 3/15 (20) | 2/15 (13) | 4/15 (27) |
|  |  | i-n13 |  | 13/14 (93) | 15/15 (100) | 13/15 (87) | 10/15 (67) | 2/15 (13) | 5/15 (33) |
|  |  |  |  |  |  |  |  |  |  |

Population averaged amplitudes and latencies (± s.d.) for oVEMP montage peaks under study. The lowermost rows show the proportion of subjects with significant responses (SR) at each stimulus intensity for the peaks contralateral (c) and ipsilateral (i) to the stimulus. Italicized and bracketed values indicate amplitudes from the SCD subject. Note: not all intensities studied are listed.
